# Atomistic evolution during the phase transition on a metastable single NaYF_4_:Yb,Er upconversion nanoparticle

**DOI:** 10.1038/s41598-018-20702-9

**Published:** 2018-02-02

**Authors:** Min Wook Pin, Eun Jin Park, Suji Choi, Yong Il Kim, Chang Hoon Jeon, Tai Hwan Ha, Young Heon Kim

**Affiliations:** 1 Korea Research Institute of Standard and Science, 267 Gajeong-ro, Yuseong-gu, Daejeon, 34113 Republic of Korea; 2 Department of Nano science, Korea University of Science and Technology (UST), 217 Gajeong-ro, Yuseong-gu, Daejeon, 34113 Republic of Korea; 3 Hazards Monitoring Research Center, Korea Research Institute of Bioscience and Biotechnology (KRIBB), 125 Gawhak-ro Yuseong-gu, Daejeon, 34141 Republic of Korea; 4 Department of Bionanotechnology, KRIBB School of Biotechnology, Korea University of Science and Technology (UST), 217 Gajeong-ro, Yuseong-gu, Daejeon, 34113 Republic of Korea; 5 Department of Materials Science and Metallurgical Engineering, Kyungpook National University, Daegu, 41566 Republic of Korea

## Abstract

The phase evolution of as-prepared NaYF_4_:Yb,Er upconversion nanoparticles (UCNPs) with a metastable cubic structure is studied based on *in situ* heating experiments via transmission electron microscopy (TEM). The atomistic behavior on the single NaYF_4_:Yb,Er UCNP is observed during the phase transition. The formation and evolution of voids on the NaYF_4_:Yb,Er UCNP appear at a temperature below 420 °C. Small circular voids are transformed at the initial stage to a large, hexagonal-pillar shaped single void. Two different routes to reach the stable α-phase from the metastable cubic structure are identified on a single NaYF_4_:Yb,Er UCNP. The first is via a stable β-phase and the second is a direct change via a liquid-like phase. The specific orientation relationships, [110]_cubic_//[11$\bar{2}$0]_hexagonal_ and {002}_cubic_//{2$\bar{2}$00}_hexagonal_, between the cubic and hexagonal structures are confirmed. Additionally, a few extra-half planes terminated in the cubic structures are also observed at the cubic/hexagonal interface.

## Introduction

NIR-to-UV/visible upconverting nanoparticles (UCNPs) have attracted much attention in biomedical applications, e.g. bioimaging, biosensors, and near infrared (NIR)-initiated drug delivery systems (DDSs). Due to a unique luminescence mechanism, UCNPs convert NIR light to ultraviolet (UV) or visible light, which can activate the photoreaction of NIR-sensitive materials. In addition, UCNPs have emerged as potential materials for various optical devices such as solid-state lasers, solar cells, flat-panel displays, and low-intensity IR imaging devices due to their attributes which include low background light and low toxicity^1–8^.

Among UCNPs, the Yb^3+^-sensitized Er^3+^ system in NaYF_4_ nanocrystalline matrices (NaYF_4_:Yb,Er) is considered to be one of the most efficient UCNPs under NIR reported to date, where NaYF_4_:Yb,Er emits green/red light under the excitation of 980 nm NIR light^3,6,7^. Er^3+^ ions are the activators (emitters) for upconversion photoluminescence and Yb^3+^ is the sensitizer in NaYF_4_. Different non-harmonic phonon coupling phenomena have been reported in two different NaYF_4_ phases (hexagonal and cubic phase structures (β and α)) which are widely used host-matrix materials for lanthanide dopants. The non-harmonic phonon mode is more severe in the cubic phase than in the hexagonal phase due to the random substitution of Na^+^ and lanthanide cations in the cubic phase, compared with the highly ordered cation distribution in the hexagonal lattice. Successful syntheses of NaYF_4_:Yb,Er nanocrystals with β- and α-phases have recently been reported based on various solution methods, e.g. modified precipitation, hydrothermal methods, and non-hydrolytic approaches. In these methods, size and shape were also controlled by adjusting the growth conditions^9–20^. The phase transition from β-phase to α-phase NaYF_4_:Yb,Er UCNPs, and as-prepared cubic structure to β-phase, was induced by heating experiments with X-ray diffraction analyses^21–23^. There is still, however, a lack of understanding of the phase behavior of NaYF_4_:Yb,Er UCNPs. Although the structural properties of NaYF_4_ have been studied for a long time, the microstructural evolution and the thermal stability on the nanometer-sized scale has not been studied well to date. An understanding of the phase stability and evolution of NaYF_4_ UCNPs is required to establish the stability and safety of these materials in order to realize bioimaging and biomedical applications. Specifically, because the α-phase NaYF_4_:Yb,Er UCNPs are considered a metastable phase at a room temperature, a detailed study on the thermal stability and the phase evolution of the UCNPs is required for more precise applications^24^.

We studied the phase evolution of NaYF_4_:Yb,Er UCNPs as a function of temperature. *In situ* heating experiments were conducted via a transmission electron microscope (TEM) to investigate the morphological and microstructural changes of NaYF_4_:Yb,Er UCNPs during heating. The atomistic evolution in a single nanoparticle is described in real time based on the analysis of high-resolution TEM images and the mechanism of the phase evolution is demonstrated in this paper.

## Experimental

NaYF_4_:Yb,Er UCNPs were synthesized via the hot-injection method. To prepare the precursor solution for an initial reaction, Y_2_O_3_ (6.832 mmol), Yb_2_O_3_ (1.750 mmol), and Er_2_O_3_ (0.182 mmol) were dissolved in aqueous trifluoroacetic acid (TFA) (50%, 70 mL) and refluxed overnight at 70 °C. After removing 5 mL of the solution containing TFA-metal complexes, it was slowly dried under vacuum at 70 °C to remove the residual water and acid. CF_3_COONa (1.25 mmol) with 2.5 mL of 1-octadecene and 5 mL of oleic acid was added into this reaction vessel. Into the second vessel, a 100 mL three-necked round bottom flask, 15 mL 1-octadecene and 10 mL oleic acid was added. The second vessel was set up with a cannula transfer to the reaction vessel containing the metal-TFA complex. Each of the solutions were heated to 125 °C under vacuum, with stirring, for 30 min to remove any residual water and oxygen. The solution in the second vessel was then heated to 310 °C and maintained under argon gas atmosphere. The solution of the reaction vessel at 125 °C was transferred dropwise into the second vessel using a syringe pump at a flow rate of 1 mL/min. After the dropwise addition was complete, the temperature of the mixture in the second vessel was lowered to 305 °C and was maintained at that temperature for 20 min under argon. It was then left to cool down to room temperature. The as-prepared materials were purified by precipitating in an excess of ethanol, centrifuging at 8000 rpm, and repeating the process. The purified materials were dispersed in *n*-hexane using ultrasonication. A shape and/or morphology of the as-prepared materials were observed by using (scanning) transmission electron microscopy (TEM). The composition of the materials were also characterized using energy-dispersive X-ray spectroscopy (EDS) analysis. The crystallinity of the as-prepared materials was evaluated using X-ray diffraction (XRD) analysis. *In situ* heating experiments in TEM were systematically conducted to study the thermal stability and phase evolution of the as-prepared materials. A microelectromechanical system (MEMS), for Fusion heating stage of Protochips Inc. (Raleigh, North Carolina), was used for the *in situ* heating experiments in TEM. The MEMS device consisted of an electrode, a heating element (silicon carbide), a membrane window (silicon nitride), and a main silicon body. For the sample preparation, the as-prepared materials were dispersed in *n*-hexane to avoid any reaction with the materials. The solution was then pipetted onto the observation area, composed of a silicon nitride membrane on the MEMS device and the solvent, *n*-hexane, was evaporated in a drying chamber at a room temperature. The MEMS device with the as-prepared materials was loaded into TEM when only the as-prepared material remained on the silicon nitride membrane after drying. The initial shape, morphology, composition, and phase were carefully observed before heating. After the observations, the materials were heated to 725 °C at a rate of 0.33 °C/s. During heating, the changes in the shape and phase were monitored and recorded as a video. All of the *in situ* experiments were conducted in the FEI Tecnai G2 F30 at 300 kV. Single crystalline NaYF_4_:Yb, Er nanoparticle image recorded at the electron-beam current density of 2.906 A/cm^2^.

## Result and Discussion

**Figure 1:**
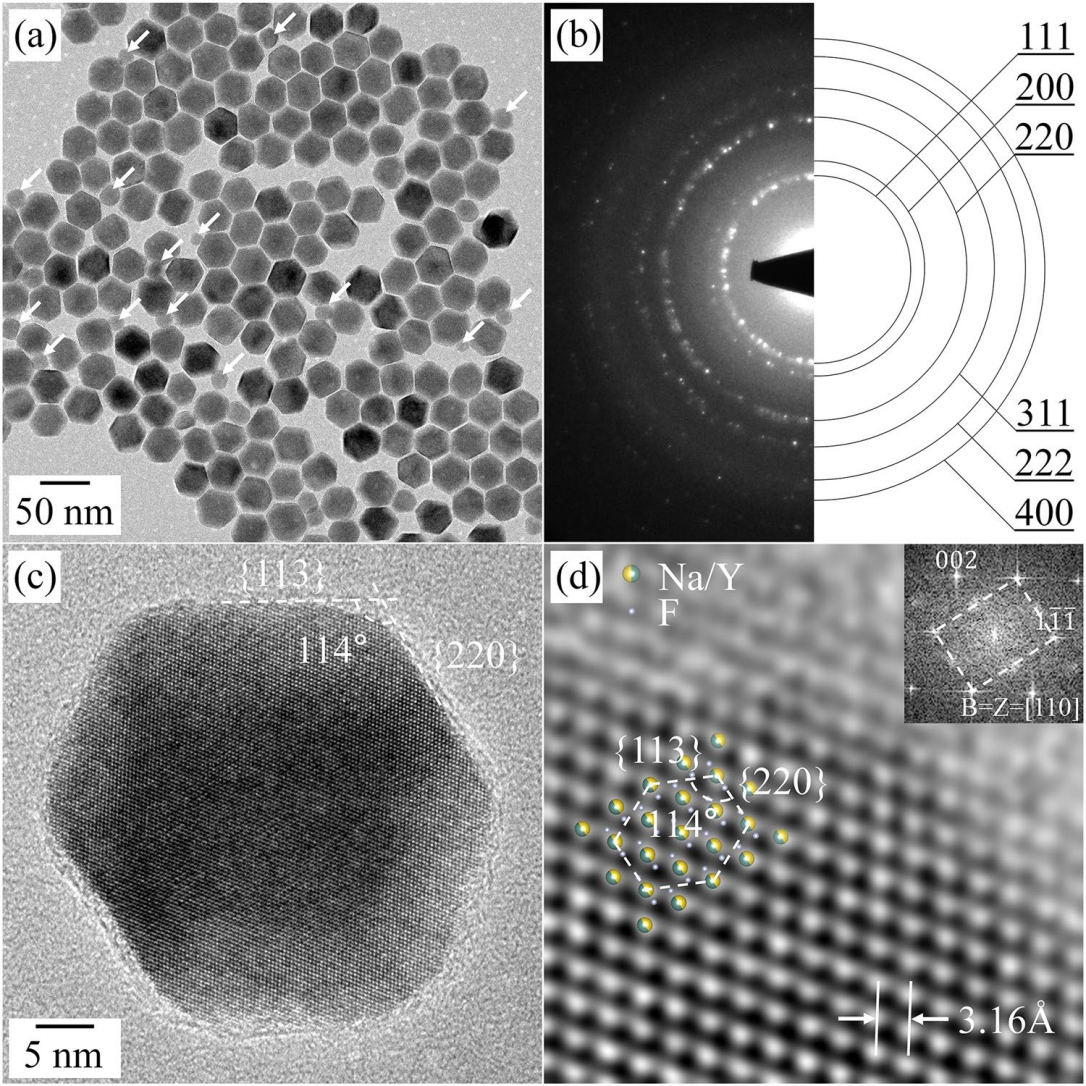
BF TEM image (**a**) and SAED pattern (**b**) of the as-prepared NaYF_4_:Yb,Er UCNPs. HR TEM (**c**) and magnified HR TEM (**d**) images of a single NaYF_4_:Yb,Er UCNP. The inset in (**d**) is the FFT result, which is indexed along the [110] direction of the cubic structure.

Figure 1(a) shows a bright-field (BF) TEM image of the as-prepared NaYF_4_-related materials. The BF TEM image indicates that the as-prepared materials are nanometer-diagonal length. Two different types of nanoparticles are observed in Fig. 1(a), one is hexagonal-pillar shaped and the other, indicated by white arrows, is spherically shaped; The hexagonal pillar shaped nanoparticles outnumber spherical ones. The diagonal lengths of nanoparticles are in the range of 38.3 ± 2.5 nm and 19.3 ± 2.3 nm for the hexagonally and spherically shaped nanoparticles, respectively (Figure S1(a)). The selected-area electron diffraction (SAED) pattern produced from the nanoparticles shows a ring pattern, which was well indexed with the cubic structure of NaYF_4_:Yb,Er (Fig. 1(b)). This result was also confirmed by the X-ray diffraction (XRD) analysis of the nanoparticles in Figure S1(b); the XRD pattern from the as-prepared materials corresponds with the standard pattern of α-phase NaYF_4_ (ICDD: 01-077-2042)^25^. In addition, the EDS spectrum from a single nanoparticles indicated that its main compositions are Na, Y, and F; the spectrum in Figure S1(c) shows 3 peaks from Na, Y, and F, respectively. The high-resolution (HR) TEM image in Fig. 1(c,d) show that the nanoparticle is a single crystalline grain. The Fast Fourier-transformation (FFT) result, inserted in Fig. 1(d), is indexed along the [110] direction of the cubic phase of NaYF_4_:Yb,Er. From the HR TEM image and the FFT result, the interplanar spacings of the {111} and {001} planes are 3.16 ± 0.04 and 5.47 ± 0.04 Å, which are consistent with those from XRD analysis.

In addition, the HR TEM image and FFT result of the small spherically shaped UCNPs indicate that they have the same cubic structure as that of the hexagonal-pillar shaped UCNPs (Figure S2). As mentioned above, most of the as-prepared nanoparticles show a hexagonal-pillar shape, except for a few small spherically shaped nanoparticles. It was difficult to detect any different morphology during repeated observations. We therefore deduced that the nanoparticles synthesized in our experiments have a hexagonal-pillar shape or near hexagonal-pillar shape, and it was further confirmed from the tilting series images from a single UCNP (Figure S3). Moreover, it was also confirmed that the NaYF_4_:Yb,Er UCNPs are surrounded by the {113} and {220} side facets of the cubic structure from the HR TEM and FFT analyses. The syntheses of hexagonal-pillar shaped cubic NaYF_4_ UCNPs have been reported by a few research groups^9,11,26^. With aids from those analyses, we can conclude that the synthesized material for further experiments is α-phase NaYF_4_:Yb,Er UCNPs with a hexagonal-pillar shape. The cubic structure NaYF_4_:Yb,Er UCNPs were slowly heated on a MEMS device, in order to study the thermal stability and the phase evolution of the UCNPs.

**Figure 2:**
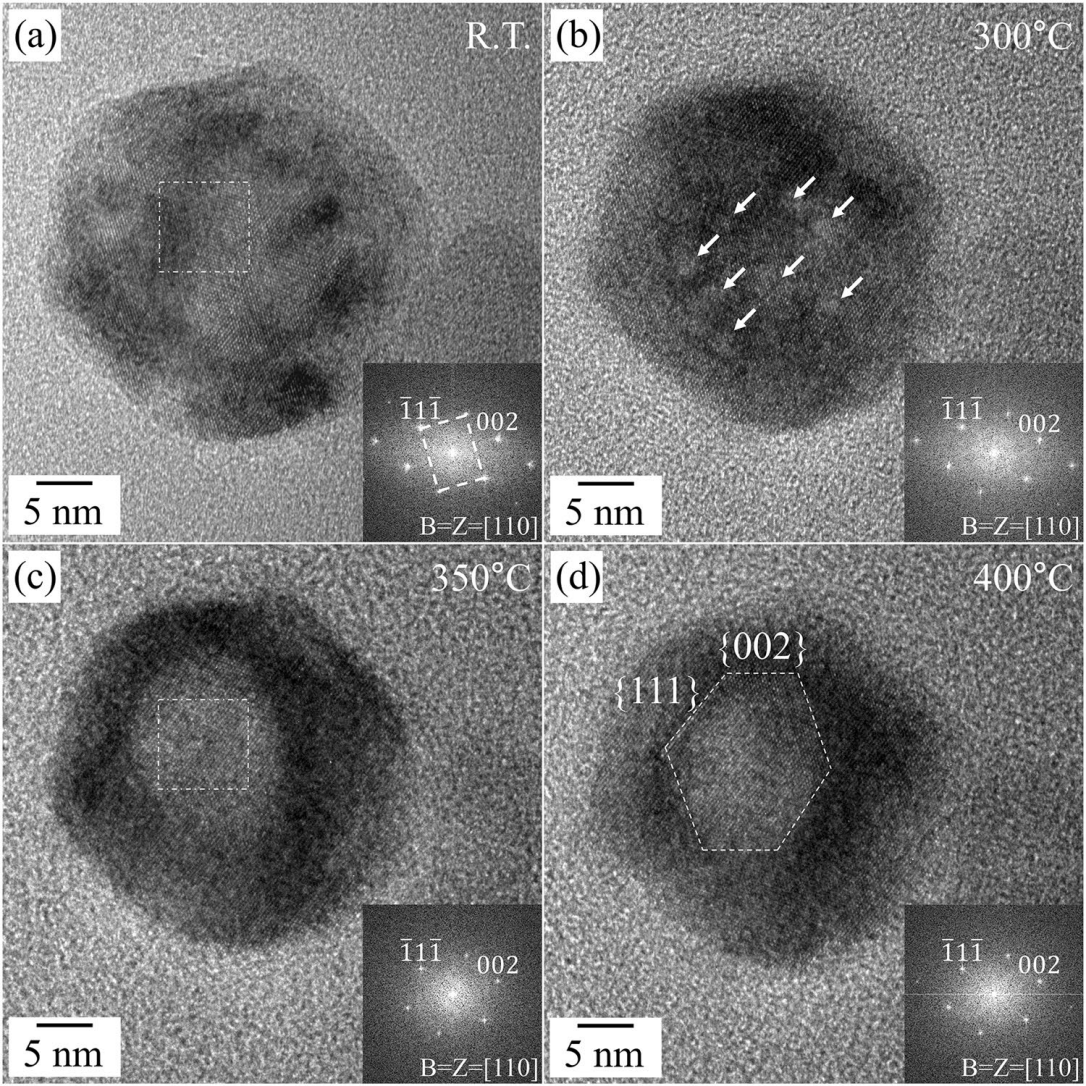
HR TEM images and corresponding FFT results of the as-prepared NaYF_4_:Yb,Er nanoparticle during the heating experiment. (**a**) Room temperature (**b**) 300 °C, (**c**) 350 °C, and, (**d**) 400 °C. All of the FFT results were indexed along the [110] direction of the cubic structure of NaYF_4_:Yb,Er.

Figure 2 shows the morphology and phase changes of the single NaYF_4_:Yb,Er UCNP, which has the same morphology and crystalline properties as the nanoparticle in Fig. 1(c) during the heating experiment from room temperature to 400 °C. With increasing temperature, a bright-and-dark contrast appeared within the UCNP, and it was observed up until approximately 300 °C. The small bright areas in Fig. 2(b), indicated by white arrows, expanded and/or agglomerated and finally unified at the center area of the UCNP with the increase in temperature (Fig. 2(c)). The bright area was then transformed into an angular polygonal shape, seen in Fig. 2(d), closely resembling a hexagonal-pillar shape. Specifically, the side facets composed of the angular-polygonal void within the UCNP were the {111} and {002} planes of the cubic structure. The diagonal length of NaYF_4_:Yb,Er UCNPs were similar before and after the formation of bright areas, while the shape of the UCNPs became slightly rounded with an increase in temperature. The bright-and-dark contrast in a TEM image is related to the atomic elements of the observing materials and the thickness along the beam direction of the samples. The atomic structure from the HR TEM images in Fig. 2 and the SAED patterns in the inset still correspond well with cubic NaYF_4_:Yb,Er, and the EDS spectra are identical before and after the formation of the bright areas (Figure S1(c,d)). It is therefore possible to deduce that the bright areas were caused by locally reducing the thickness of the sample. The local reduction in the thickness of the NaYF_4_:Yb,Er UCNPs forms voids on the surface and/or inside the nanoparticles. The formation of voids in NaYF_4_:Yb,Er UCNPs is similar to the behavior of hollow formation in UCNPs by electron beams, reported by Feng *et al*.^27^. It is suggested that the existence of organic materials capping NaYF_4_:Yb,Er UCNPs can trigger formation of the hollow in the UCNPs^28^. This was experimentally confirmed on the surface of the nanoparticles (Figure S4.), as described above. In addition, the change of the contrast in the TEM images could be caused by the rearrangement of the lattice defects by thermal activation^29,30^. In actuality, the polygonal-shaped contrast in the α-phase NaYF_4_:Yb,Er UCNPs is not explained by the pure electron-beam effect. The formation of hexagonal-like contrasts may be related not only to the electron-beam effect, but also to the thermal effect. The UCNPs must be thermodynamically stabilized by lowering the total energy by forming low-index {111} and {002} planes composed of voids with low surface energies at higher temperatures. Surface diffusion and migration are more enhanced by the electron beam and thermal energy from underlying heating stage.

**Figure 3:**
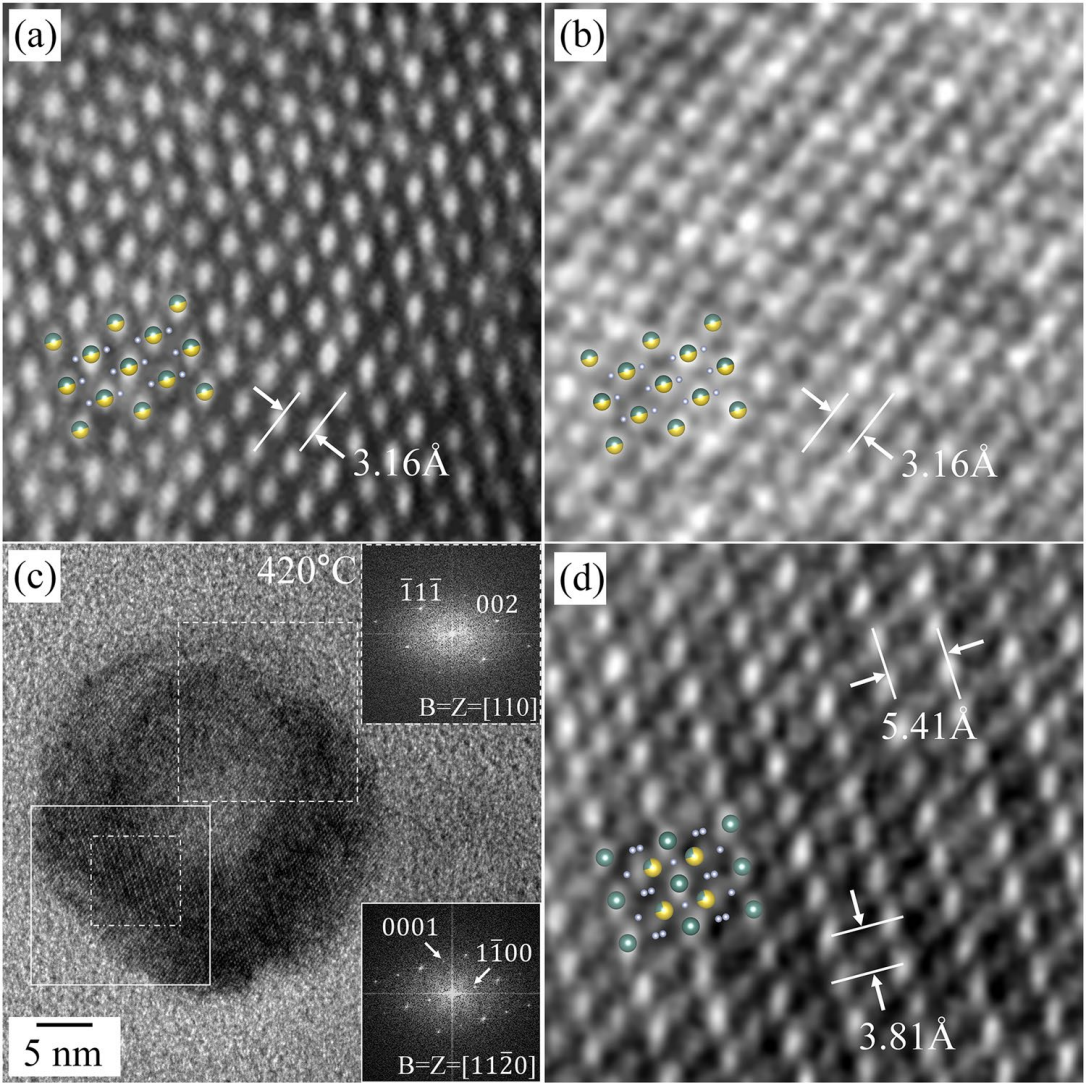
Magnified HR TEM images of the NaYF_4_:Yb,Er UCNPs showing the atomic structure during heating. (**a**) Room temperature (Rectangle in Fig. 2(a)), (**b**) 400 °C (Rectangle in Fig. 2(b)), (**c**) HR TEM image at 420 °C, (**d**) 420 °C (Rectangle in Fig. 3(c)). The FFT results in the top and bottom insets in (**c**) were indexed along the [110] and [11$\bar{2}$0] directions of the cubic and hexagonal structures, respectively.

The HR TEM and magnified HR TEM images in Fig. 3 show the atomic structure at room temperature, 400 °C, and 420 °C, respectively. The HR TEM image of the as-prepared NaYF_4_:Yb,Er UCNP shows a well-ordered atomic structure of the cubic structure, the interplanar spacings correlate well with the cubic structure (Fig. 3(a), magnified from the rectangle in Fig. 2(a)). Despite of the formation of the void at 400 °C, the UCNP was free from any structural change; the atomic structure of the void area is the same as that of the as-prepared nanoparticle (Fig. 3(b), magnified from the void region in Fig. 2(c)). However, a specific atomic arrangement emerged when the heating temperature reached 420 °C. The HR TEM image captured at 420 °C, shown in Fig. 3(c), shows the UCNP with a complex contrast; the top area is brighter while the bottom area is darker, compared with Fig. 2(d). The FFT result in the top inset was taken by FFT of the dashed rectangular area, and is completely indexed to have [110] zone axis with the initial cubic structure (Fig. 3(c)). On the other hand, a few extra diffraction spots indicated by arrows are newly emerging in the FFT result taken from the straight rectangle (the bottom inset of Fig. 3(c)); specifically, a spot is located near a half distance of the 002 spot from the cubic structure. In the magnified HR TEM image of the straight rectangular area, the atomic arrangement is totally different from that in the cubic structure; the lattice planes with a large interplanar spacing, approximately 5.41 ± 0.04 Å, are observed in this area, which is consistent with the existence of a diffraction spot near the half distance of the 002 spot of the cubic structure (Fig. 3(c)). From the analyses of atomic arrangements, it could be concluded that the hexagonal phase (β-phase) of NaYF_4_:Yb,Er emerges at a temperature of 420 °C. As revealed in detail later, it was confirmed that the orientation relationship of [110]_cubic_//[11$\bar{2}$0]_β_ and {002}_cubic_//{2$\bar{2}$00}_β_, exists between the cubic and hexagonal structures.

**Figure 4:**
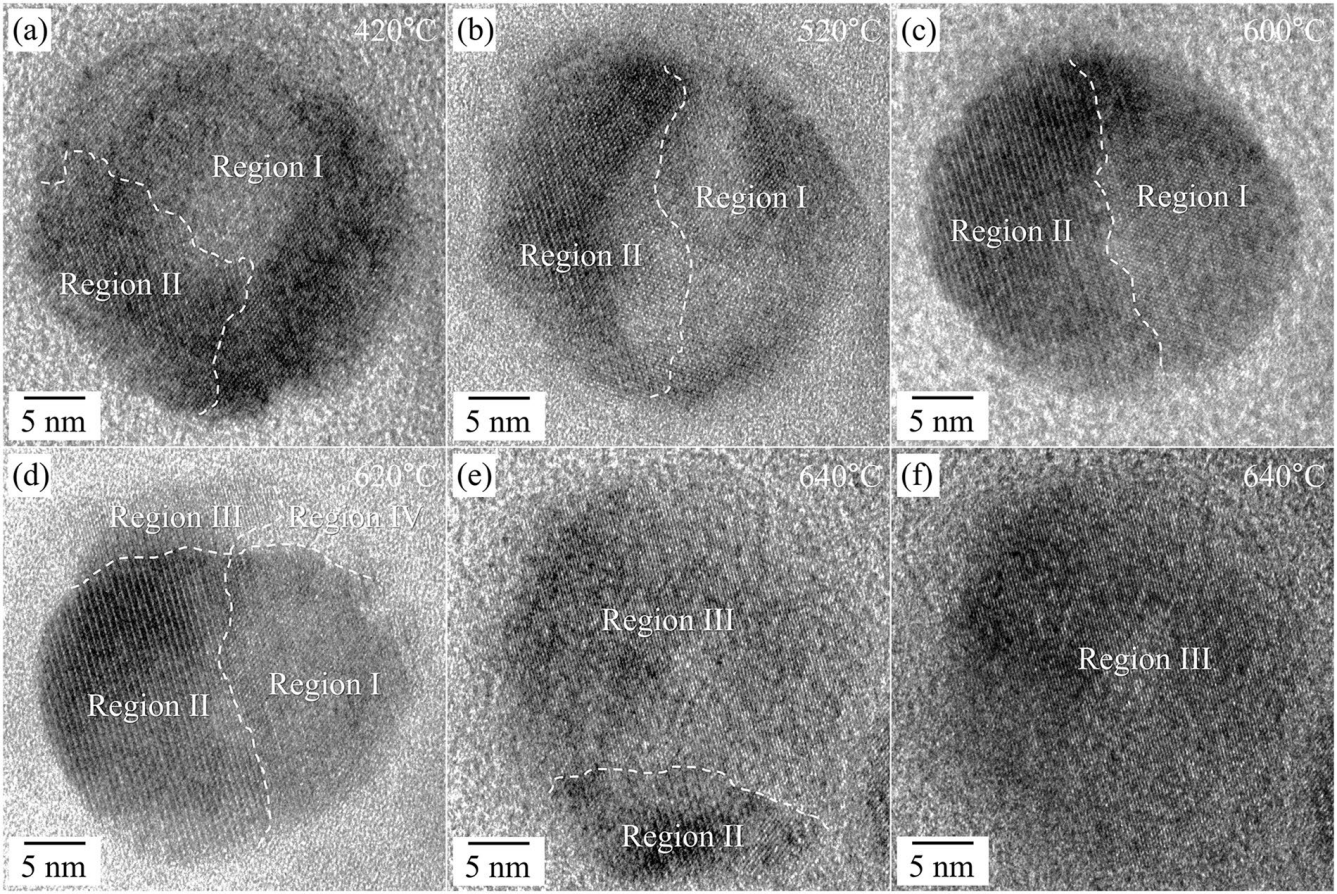
HR TEM images of the NaYF_4_:Yb,Er UCNPs during heating experiments at the higher temperature (420 °C–640 °C). (**a**) 420 °C, (**b**) 520 °C, (**c**) 600 °C, (**d**) 620 °C, and (**e**,**f**) 640 °C. The dotted lines indicate the boundaries between the different phases (Region I-the metastable cubic phase, Region II-the hexagonal β-phase, Region III-the stable α-phase, and Region IV-liquid-like phase).

The shape and the atomic arrangement changed continuously during the heating process over 420 °C. The hexagonal-pillar shape of as-prepared NaYF_4_:Yb,Er UCNPs was somewhat rounded at the apexes upon increasing the temperature (Fig. 4).

The region of β-phase NaYF_4_:Yb,Er, first observed at 420 °C, gradually encroached the territory of the cubic phase (α-phase) upon increasing the heating temperature to 600 °C (Region II in Fig. 4(a–c)). However, a large portion of the single UCNP remained as the metastable cubic structure (Region I in Fig. 4(a–c)). By further increasing the temperature above 620 °C, the quantity of the β-phase dramatically diminished (at 640 °C), whilst it was stable at a maintained temperature of 620 °C (Fig. 4(d–f) and Figure S5). Eventually, the NaYF_4_:Yb,Er UCNP was completely transformed from the as-prepared state (the metastable cubic structure) to the stable cubic one (Region III in Fig. 4(e,f)) via the intermediate hexagonal structure at 640 °C (Region II). We therefore deduced that the transition from the β-phase to the stable α-phase occurs through a thermal activation process.

However, it is noteworthy that the phase transition in the remained area (Region I, maintaining the initial metastable cubic phase), is different to that of the area transformed to β-phase. As shown in Fig. 4(d) and Figure S7, the NaYF_4_:Yb,Er UCNP could be divided into four areas with different crystallinities: Region I, the dark area in the bottom right, represents the metastable cubic structure region of the as-prepared state, Region II, the darkest area in the bottom left represents the β-phase, Region III, the bright area in the top left represents the stable new cubic phase, and Region IV, the brightest area in the top right represents the amorphous (or liquid-like) phase. Recalling that the lowest eutectic temperature is 638 °C in the NaF-YF_3_ binary phase diagram^31,32^, we therefore conjectured that a liquid-like phase is involved for the high-temperature phase transformation near 620 °C and the rapid extinction of the β-phase can be explained by the existence of the liquid-like phase. Figure S7 shows the details of the phase transformation as a function of time at 620 °C. Region I, with the metastable cubic structure, changed gradually to the stable α-phase with time at a fixed temperature of 620 °C. Specifically, the areas with stable α-phases expanded near the liquid-like region and all areas with metastable cubic structures changed completely to stable α-phases, as shown in Figure S7(c). The FFT result from Region I with the newly emerging crystal phase is the same as that from Region I as part of the as-prepared nanoparticle, indicating that the structure of the new phase is the same as the crystal structure of the as-prepared nanoparticle; the positions of the FFT spots are identical in the two FFT results. From these observations, it is concluded that the as-prepared NaYF_4_:Yb,Er UCNPs were a metastable phase with a cubic structure (metastable α-phase) and were transformed to a stable cubic one (stable α-phase) by thermal treatment. After some time at the annealing temperature of 620 °C, the metastable cubic structure was more rapidly transformed to the stable α-phase via liquid-like areas, while the phase transition from the β-phase to the stable α-phase were concomitantly activated. Apparently, the liquid-like area accelerates the phase transformation; the existence of Moiré fringes on the area with the metastable cubic structure may implicate this process (Figure S7(a,b)). The SAED pattern taken at a high temperature, 725 °C, also fortifies the phase transformation to the stable cubic phase of NaYF_4_:Yb,Er UCNPs (Figure S8).

**Figure 5:**
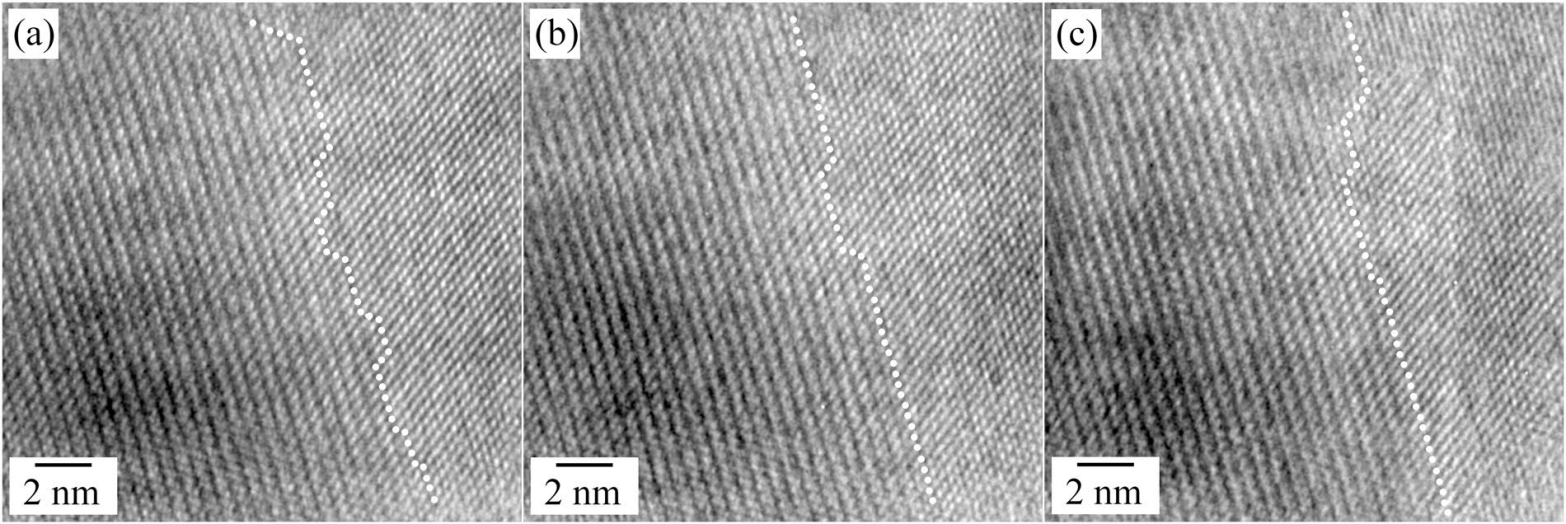
HR TEM images taken during the phase transformation from the metastable cubic phase to the hexagonal phase of the NaYF_4_:Yb,Er UCNPs (time interval: 1 min, temperature: 620 °C). The dots indicated the termination of the hexagonal phase (right side: metastable cubic phase, left side: hexagonal phase).

The changes of the atomic structure during the phase transformation from the metastable cubic phase to the hexagonal one are shown in Fig. 5. In the magnified HRTEM images, the interface between the metastable cubic structure and the hexagonal structure is composed with flat ledges and a few steps; the phase change was continued along the [1$\bar{1}$00] direction of the hexagonal structure and the [002] direction of the cubic structure and by the continuous formation of steps and the continuous filling of the gaps between steps (Fig. 5(a,b)). The ledges were composed with the {1$\bar{1}$00} planes and the steps were slanted kinks, close to the {1$\bar{1}$02} planes of the hexagonal structure. Because this kind of the phase transformation initiated at a low temperature, the composition is conserved with that of the parent phase since the diffusion is limited in a narrow area. The rearrangement of the atoms during the phase transformation may be easily achieved by a small driving force because the parent phase is metastable. From these observations, we deduced that the phase transformation process from the metastable cubic structure to the hexagonal structure is similar to that of the massive transformation^33,34^.

**Figure 6:**
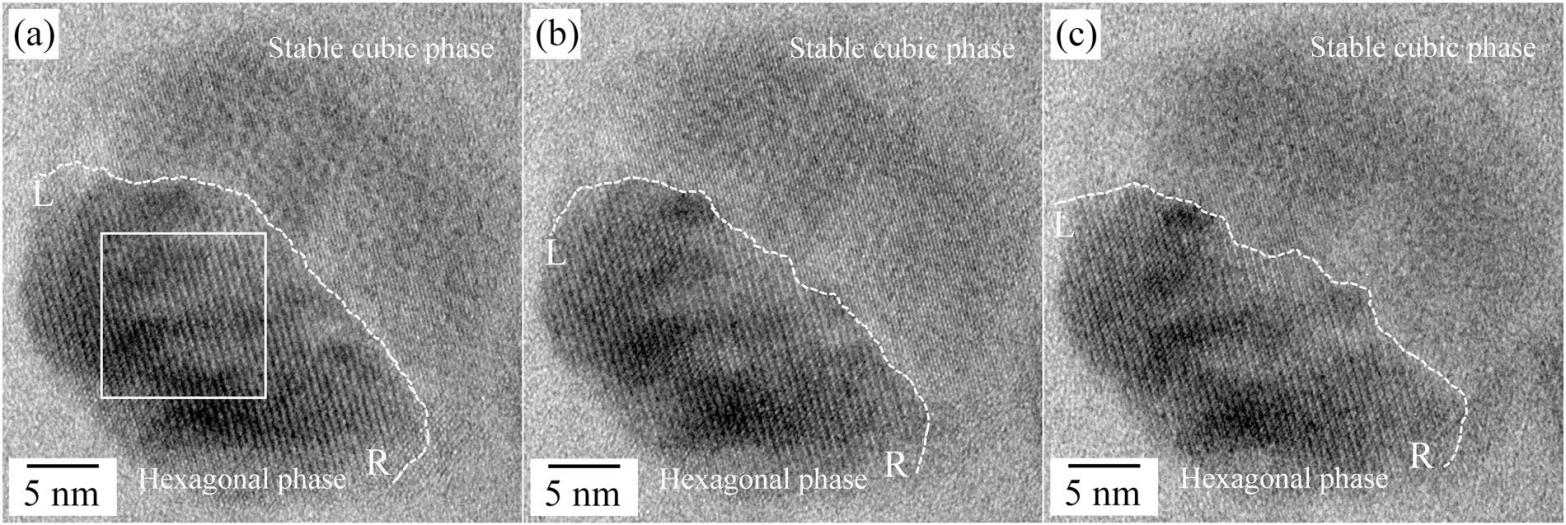
HR TEM images taken during the phase transformation from the hexagonal phase to the stable cubic phase of the NaYF_4_:Yb,Er UCNPs (time interval: 1 min, temperature: 620 °C).

Next, the atomic evolution during the phase transformation from the hexagonal structure to the stable cubic structure was also detailed and shown in Fig. 6. The phase transformation from the hexagonal phase to the stable cubic phase was mainly progressed at the interface between the hexagonal/stable cubic phases which had been formed from the metastable cubic phase. In the HRTEM images, the boundaries between two phases, the hexagonal and the stable cubic phases, were continuously changed from the left end (L) to the right end (R) with time and a certain part of the boundaries was not abrupt. A contrast modulation was observed inside the hexagonal phase as indicated by straight rectangle in Fig. 6(a), suggesting the possibility of the existence of the liquid-like phase, as demonstrated in Fig. 4. Because the phase transformation from the hexagonal phase to the stable cubic phase was conducted at a high temperature (over 620 °C), involvement of liquid-like phase seems quite plausible. The liquid-like phase was rapidly moving on the NaYF_4_:Yb,Er UCNPs UCNPs (Movie M1). In addition, severe changes of the curvature for the center of the hexagonal phase were observed near both ends of the boundaries, L and R. This behavior may be related to the size effect of the NaYF_4_:Yb,Er UCNPs, e.g. surface melting. From these observations, we concluded that the phase transformation was contributed by the diffusion; the stable cubic phase continuously grew while conserving the epitaxial relationship with the parent hexagonal phase.

**Figure 7:**
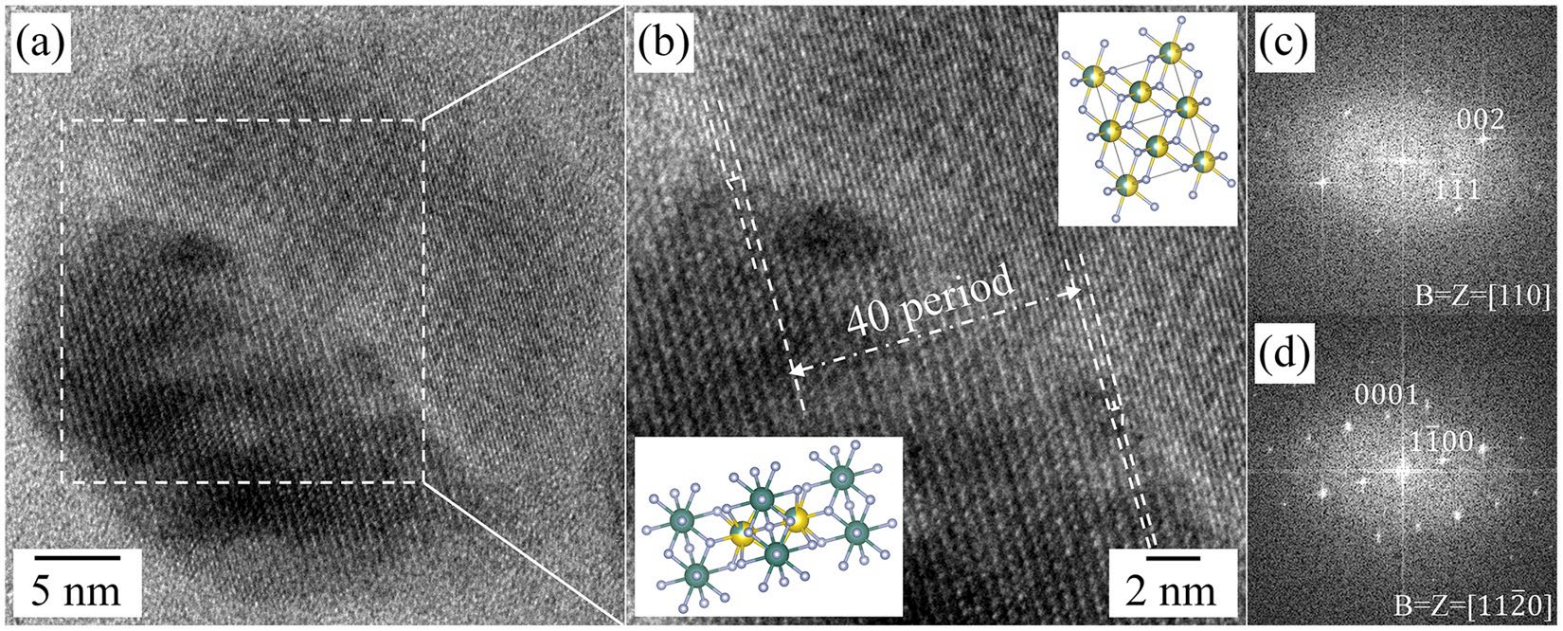
(**a**) Atomic arrangements at the stable cubic/hexagonal interface at 620 °C in a single NaYF_4_:Yb,Er UCNP during the heating experiment. (**b**) Magnified HR TEM image taken from the dotted rectangle in (**a**); the dislocation symbols indicate the extra-half planes terminated in the stable cubic α-phase observed at the interface. The top and bottom insets show the atomic models of the stable cubic α and hexagonal β phases, respectively. (**c**,**d**) FFT results taken from the stable cubic α-phase and β-phase, respectively; the FFT results were indexed along the [110] and [11$\bar{2}$0] directions of the cubic and hexagonal structures, respectively.

Although a liquid-like phase is introduced for the phase transformation, specific orientation relationships could be found between the hexagonal β-phase and the metastable/stable cubic α-phase: [110]_cubic_//[11$\bar{2}$0]_hexagonal_ and {002}_cubic_//{2$\bar{2}$00}_hexagonal_ (Fig. 7(c,d)). The HR TEM image in Fig. 7(a) shows the atomic arrangement at the cubic/hexagonal interface. Extra-half planes terminated in the cubic structure are observed at the interface, indicated by edge dislocation symbols (Fig. 7(b)). In the case of the specific orientation relationship, the misfit between the cubic and hexagonal structures, *δ*, is defined by

1 $$\delta =\frac{({d}_{2200,hexa}-2\times {d}_{002,cub})}{({d}_{2200,hexa})}\cong 0.060$$

where d_2200,hexa_ and d_002,cub_ are the interplanar spacings of the {2$\bar{2}$00} planes of the hexagonal structure and the {002} planes of the cubic structures, respectively. If extra-half planes are introduced into the structure for strain relaxation, the average spacing *S* of misfit planes depends on the misfit and is of the order of *d*_*002,cub*_*/δ*. Therefore the *S* is determined as follows:

2 $$s=\frac{(2.735)}{(0.060)}=45.58\,\AA $$

Because the interplanar spacings of 2$\bar{2}$00 planes of the hexagonal structure and that of 002 planes of the cubic structure are about 2.580 Å and 2.735 Å, the $\bar{2}$200 plane of the hexagonal structure and the 002 plane of the cubic structure must accord with each other about every 17 planes for the hexagonal structure and 16 planes for the cubic structure. The spacing between two extra-half planes in the cubic structure is experimentally approximated to 106.0 Å, which indicates that there are much less extra-half planes than the theoretical value at the interface between the hexagonal and cubic structures. Specifically, moreover, the extra-half planes were observed in the cubic structure, not in the hexagonal structure. This specific atomic structure at the interface may be caused by the complex phase evolution during heating, e.g. the coexistence of β-phase and liquid-like phase. Collective atomic structures at the interface were depicted in Figure S8 to help one envision the β- to α-phase transformation, based on the experimental observations. The strains along the in-plane directions, [002] and [1$\bar{1}$0] directions of the cubic structure, are asymmetric at the interface; the lattice mismatch along the [110] direction is larger than that along the [001] direction (Figure S8). Since, the atomic rearrangements for the phase transformation would be severely affected by the strain relaxation behavior, according to the atomic diffusion with minimal atomic displacements and their intermixing. In addition, the strain behavior in nanometer-sized materials is also affected by the surface. A more detailed study is required to understand the phenomena at the interface.

**Figure 8:**
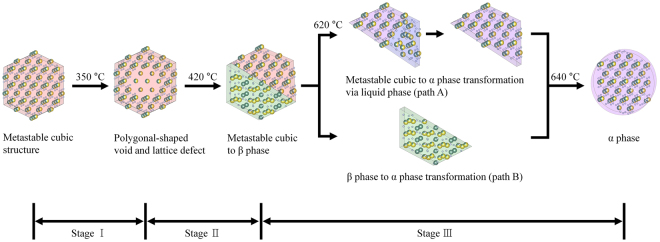
Schematic illustration of the phase transformation of α-phase NaYF_4_:Yb,Er nanoparticle from metastable cubic structure NaYF_4_:Yb,Er nanoparticle.

In Fig. 8, a schematic illustration is shown to summarize the whole phase transitions from metastable cubic structure to stable cubic phase (α-phase) transformation, encompassing three distinct stages^35^. The first stage is the formation of polygonal-shaped void, which might be caused by surface rearrangements possibly caused by thermal activation and some enhancement from electron beam (as shown in Fig. 3). The second stage is the emergence of hexagonal phase (β-phase) of NaYF_4_:Yb,Er around 420° (as shown in Fig. 4), and almost half of the UCNP nanocrystal on average transformed to the hexagonal phase. The orientation relationship between metastable cubic structure and β- phase are revealed to be [110]_cubic_//[11$\bar{2}$0]_β_ and {002}_cubic_//{2$\bar{2}$00}_β_. The last stage consists of two different routes to stable α-phase transformation from metastable α-phase via liquid phase (*path A*), and from β-phase by thermally activated atomistic diffusion process (*path B*). Considering the eutectic point of NaYF_4_ (638 °C), both paths (at 620 °C ~ 640 °C) appear to have a relation with amorphous liquid-like phase.

## Conclusion

We have studied the phase evolution of as-prepared NaYF_4_:Yb,Er UCNPs with a metastable cubic structure. The formation of voids in the NaYF_4_:Yb,Er UCNPs was observed at a minimum temperature of 420 °C, above which the small circular voids at the initial stage finally merged into hexagonal-pillar shaped larger voids. On a single NaYF_4_:Yb,Er UCNP, two different routes were identified to reach the stable α-phase from the metastable cubic structure of as-prepared NaYF_4_:Yb,Er UCNPs. The first is via the stable β-phase by heating, and the other is through the direct change from the metastable structure to the stable phase. The stable β-phase of NaYF_4_:Yb,Er partially emerged at 420 °C, and it was again transformed to the stable α-phase over 620 °C. At this temperature, the remaining metastable cubic phase in the single nanoparticle was directly transformed to the stable α-phase. The existence of a liquid-like phase was identified over 620 °C, and the specific orientation relationship, [110]_cubic_//[11$\bar{2}$0]_hexagonal_ and {002}_cubic_//{2$\bar{2}$00}_hexagonal_, was confirmed between the cubic and hexagonal structures. Additionally, a few extra-half planes terminated in the cubic structure were observed at the cubic/hexagonal interface.

## Electronic supplementary material

Supplementary Information
