# Supplementary material for: Atomistic evolution during the phase transition on a metastable single NaYF4:Yb,Er upconversion nanoparticle
Source: Sci Rep. 2018 Feb 2;8:2199. doi: 10.1038/s41598-018-20702-9 (PMC5797087; doi:10.1038/s41598-018-20702-9)
Supplement: Supplementary file 1 — Supplementary Information [file 41598_2018_20702_MOESM1_ESM.pdf]

Supplementary information

Atomistic evolution during the phase transition on a metastable single $NaYF_4$:Yb,Er upconversion nanoparticle

Min Wook Pin[1,2,+], Eun Jin Park[3,4,+], Suji Choi[1,5], Yong Il Kim[1,2], Chang Hoon Jeon[3,4], Tai Hwan Ha[3,4,*], Young Heon Kim[1,2,*]

[1]Korea Research Institute of Standard and Science, 267 Gajeong-ro, Yuseong-gu, Daejeon 14113, Republic of Korea.

[2]Department of Nano science, Korea University of Science and Technology (UST), 217 Gajeong-ro, Yuseong-gu, Daejeon 34113, Republic of Korea.

[3]Hazards Monitoring Research Center, Korea Research Institute of Bioscience and Biotechnology (KRIBB), 125 Gawhak-ro Yuseong-gu Daejeon 34141, Republic of Korea.

[4]Department of Bionanotechnology, KRIBB School of Biotechnology, Korea University of Science and Technology (UST), 217 Gajeong-ro, Yuseong-gu, Daejeon 34113, Republic of Korea.

[5]Department of Materials Science and Metallurgical Engineering, Kyungpook National University, Daegu 41566, Republic of Korea.

Image filtering

Figure 1(d), Figure 3(a), (b) and (d) has been filtered using ABSF (Average Background Subtracted Filter) this filter is available as a script for digital micrographs (D. R. G. Mitchell, v2.0, January 2014). The parameters for ABSF were Delta= 2 %, BWn= 3 (order of Butterworth filter) and BWRo= 0.5 (radius of Butterworth filter in pixels).

Method details

Y_2O_3 (99.99%) and Yb_2O_3 (99.9%) were purchased from Sigma-Aldrich (China), Er_2O_3 (99.99%), sodium trifluoroacetate (98%), 1-octadecene (90%) were purchased from Sigma-Aldrich (USA), and ammonium fluoride (98%), trifluoroacetic acid (98%) were purchased from Sigma-Aldrich (Germany) while oleic acid (85%) was obtained from TCI (Japan).

Fast Fourier Transformation (FFT) Result Accuracy

The resolution of FFT image was 0.055 nm^{-1}/pixel in our analysis. The distance measured in the FFT is the inverse of a number in real space. We assumed that the accuracy of the measurement is related to the decision of spot positions in FFT results. Since, the accuracy was calculated to approximately 0.082 Å by considering the spreading of the $\{1\bar{1}00)$ inter-planar spacing of the hexagonal structure.

FIGURES

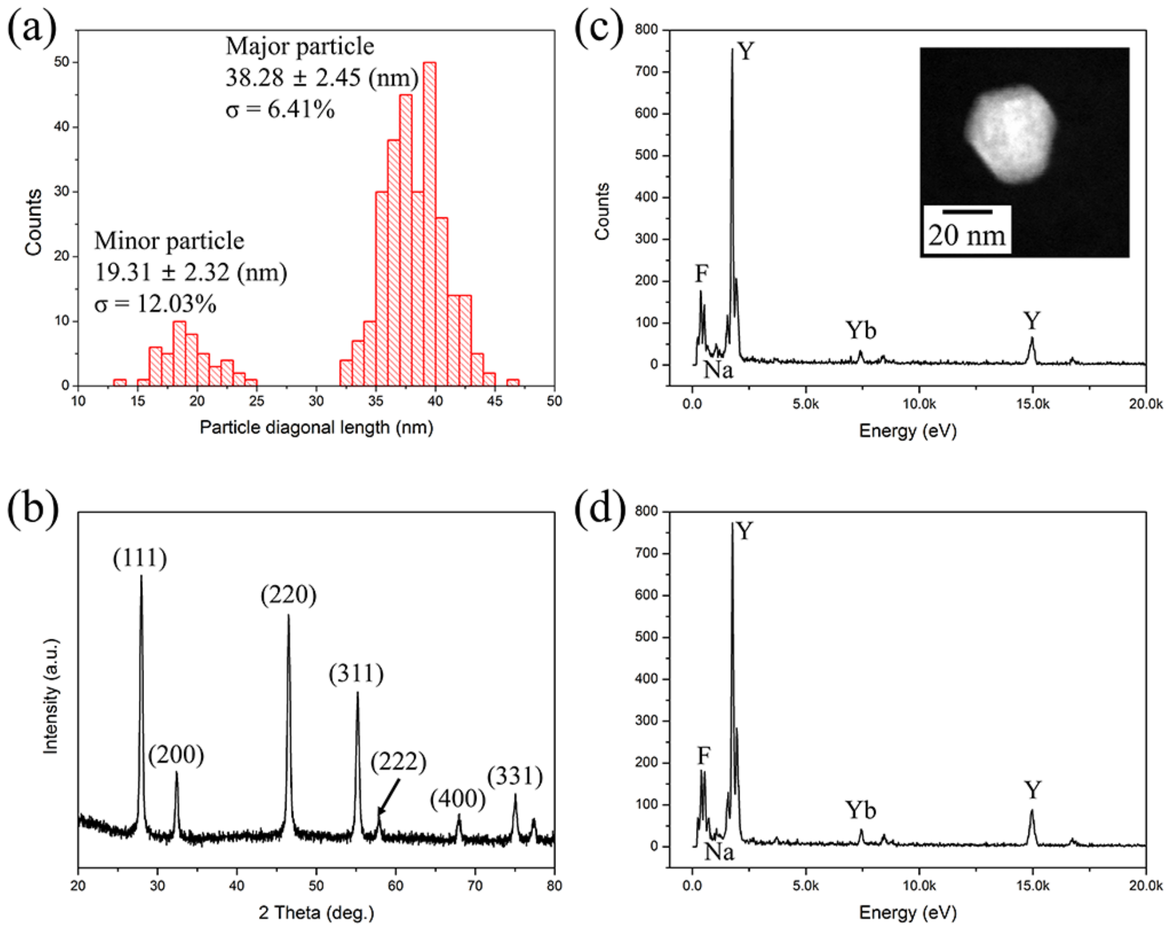

Figure S1. Histogram of size distribution obtained from TEM images (a) and (b) XRD spectra of as-prepared $NaYF_4$:Yb,Er nanoparticles. EDS spectra of in situ heating experiment for $NaYF_4$:Yb,Er nanoparticle before (c) and after (b) heating.

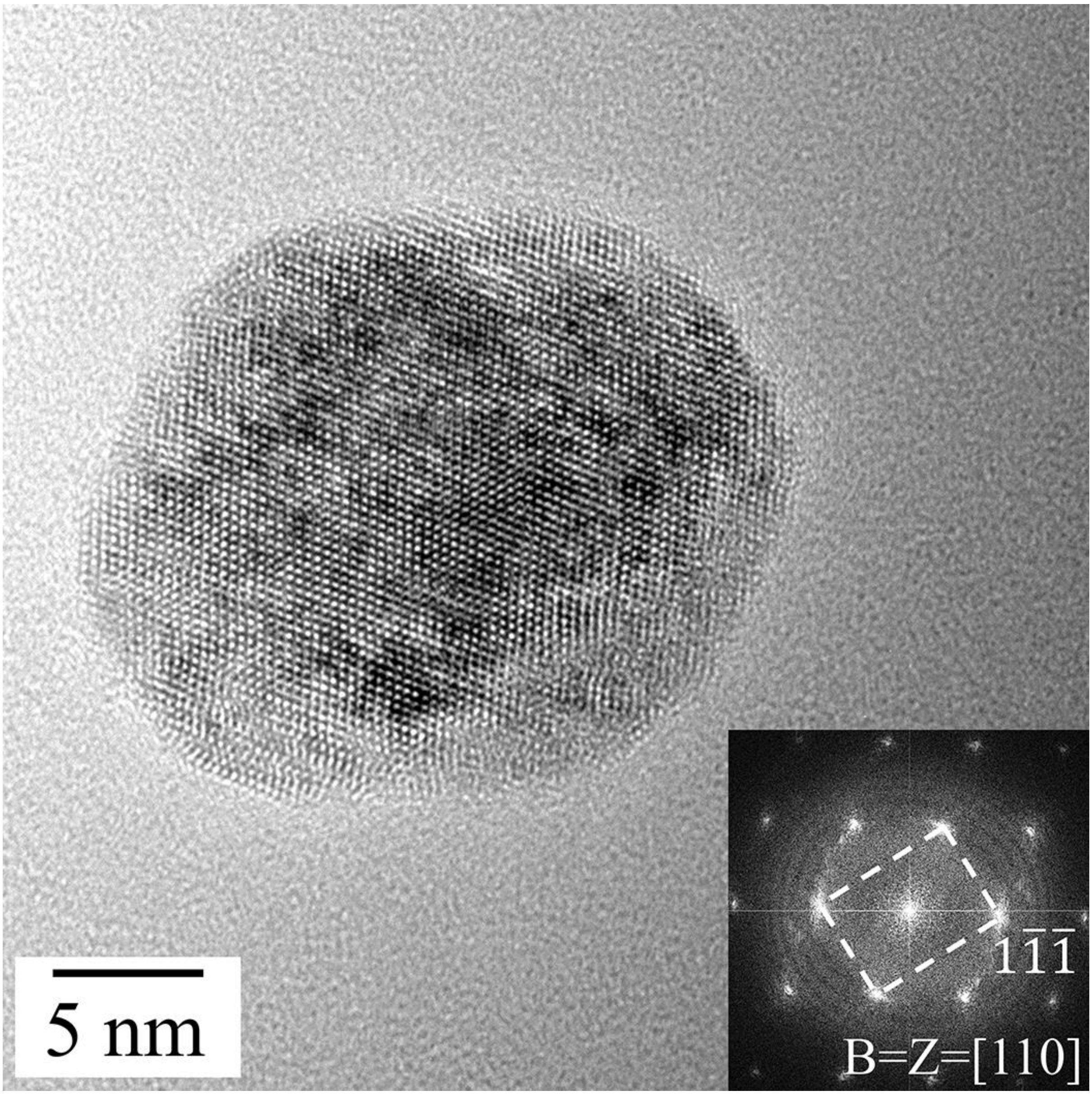

Figure S2. HR TEM image of the small spherically shaped $NaYF_4$:Yb,Er nanoparticles from Figure 1 (a), indicated by white arrows. The inset is the diffractogram by FFT, which is indexed along the [110] direction of the cubic structure.

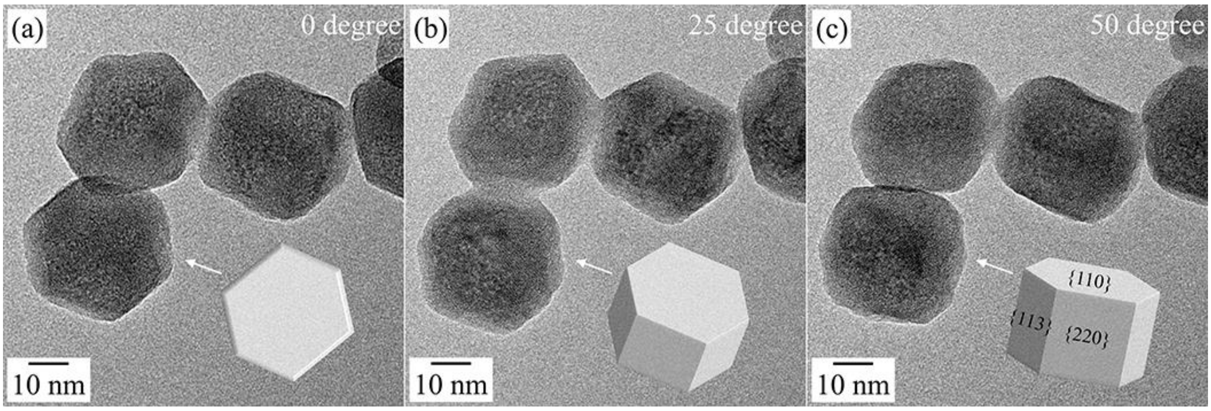

Figure S3. BF TEM image of the hexagonally shaped $NaYF_4$:Yb,Er nanoparticles observed with 0 degree (a), tilting for 25 degree (b), and tilting for 50 degree (c). The hexagonal plate illustration included for each tilting series of the TEM images, is indicated by white arrows.

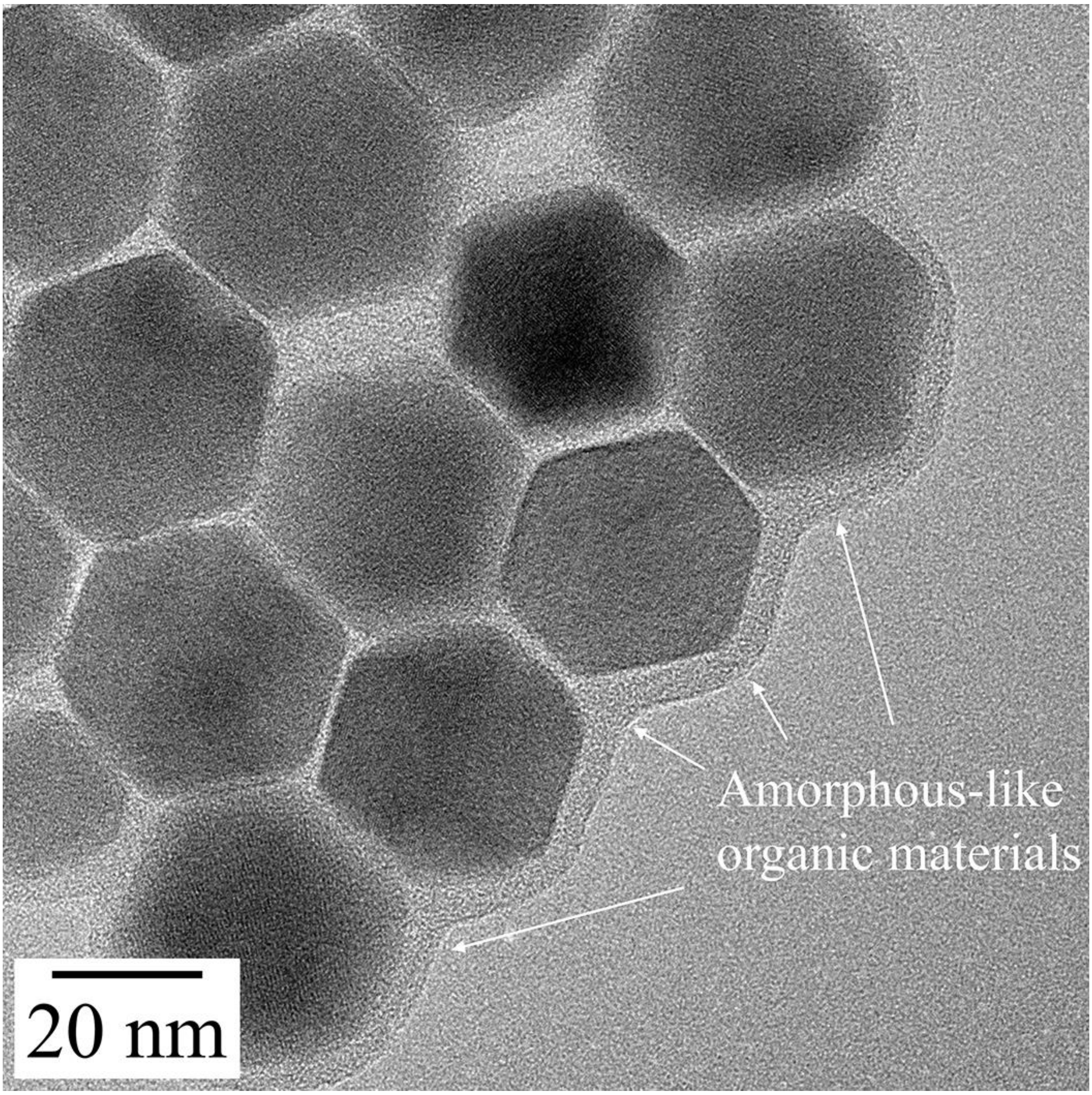

Figure S4. BF TEM image of $NaYF_4$:Yb,Er nanoparticles engulfed in an amorphous-like organic materials, indicated by white arrows.

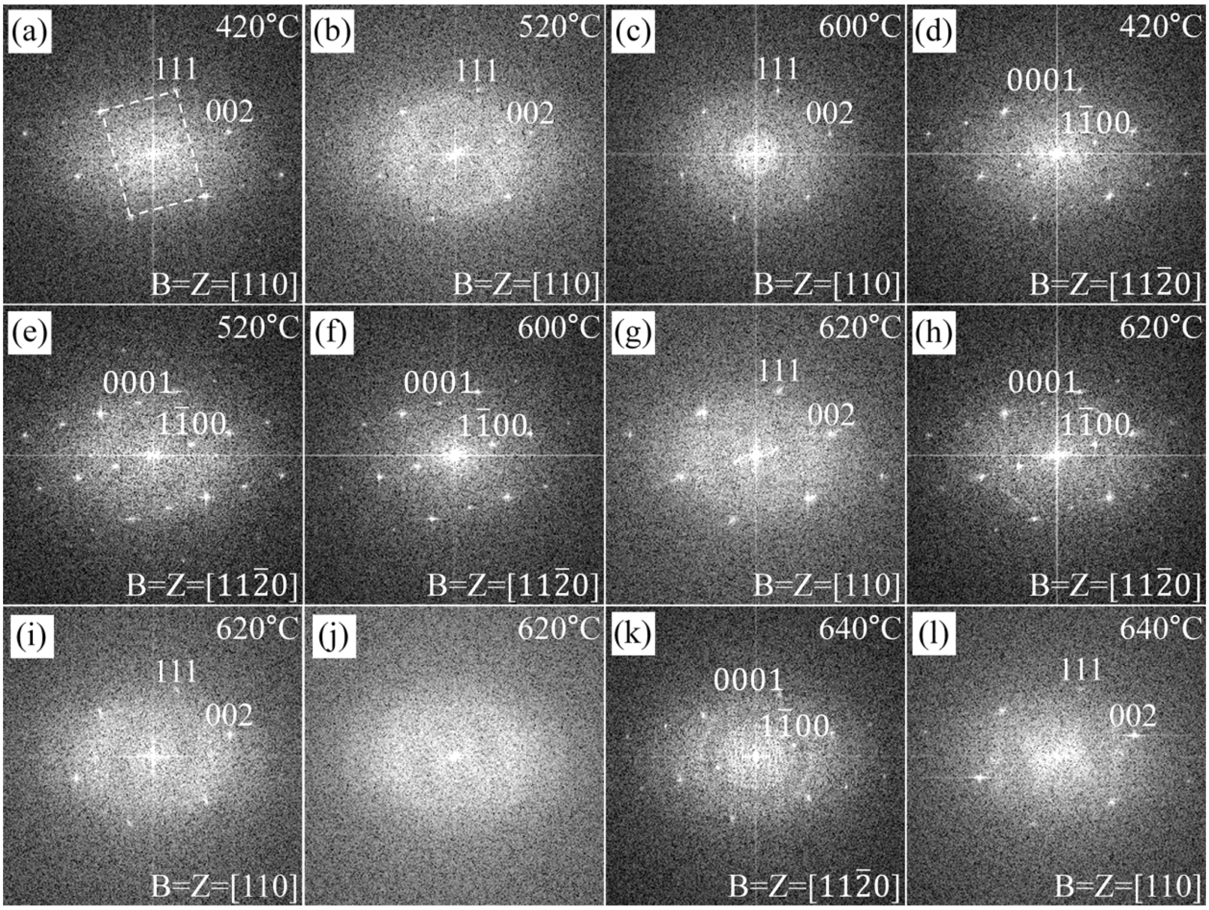

Figure S5. Diffractogram by FFT for the structural evolution of $NaYF_4$:Yb,Er nanoparticles from Figure 4. (a-c) and (d-f) from Figure 4 (a-c) in region I and region II, respectively. (g-j) from Figure 4 (d) in region I, II, III, and IV, respectively. (k-l) from Figure 4 (e) region II and III. The heating temperature included each images.

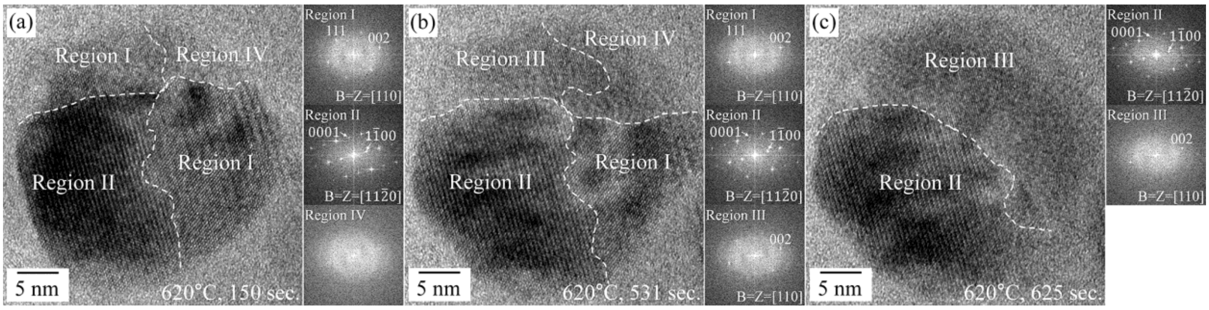

Figure S6. Time dependent HR TEM image of $NaYF_4$:Yb,Er nanoparticles at 620 °C during the heating experiment. (a) 150 s, (b) 531 s, and (c) 625 s. The diffractogram by FFT are included insets for each time series of HR TEM images. The areas with different phases were divided by white dashed lines.

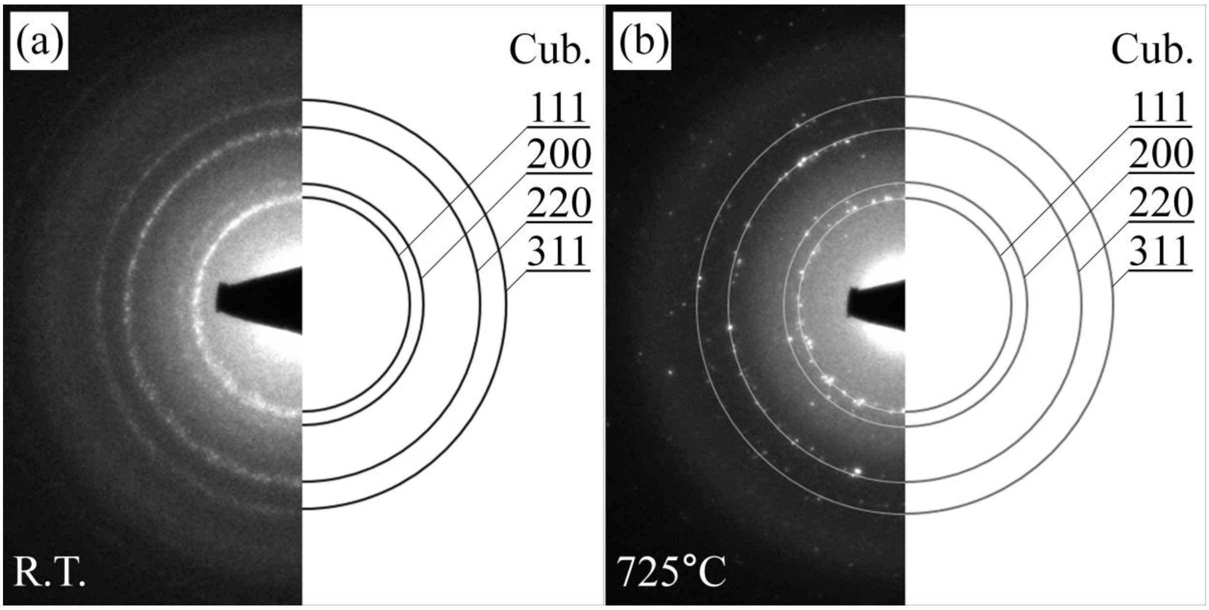

Figure S7. SAED patterns taken from $NaYF_4$:Yb, Er UCNPs during the heat experiment. (a) R.T. and (b) 725 ℃.

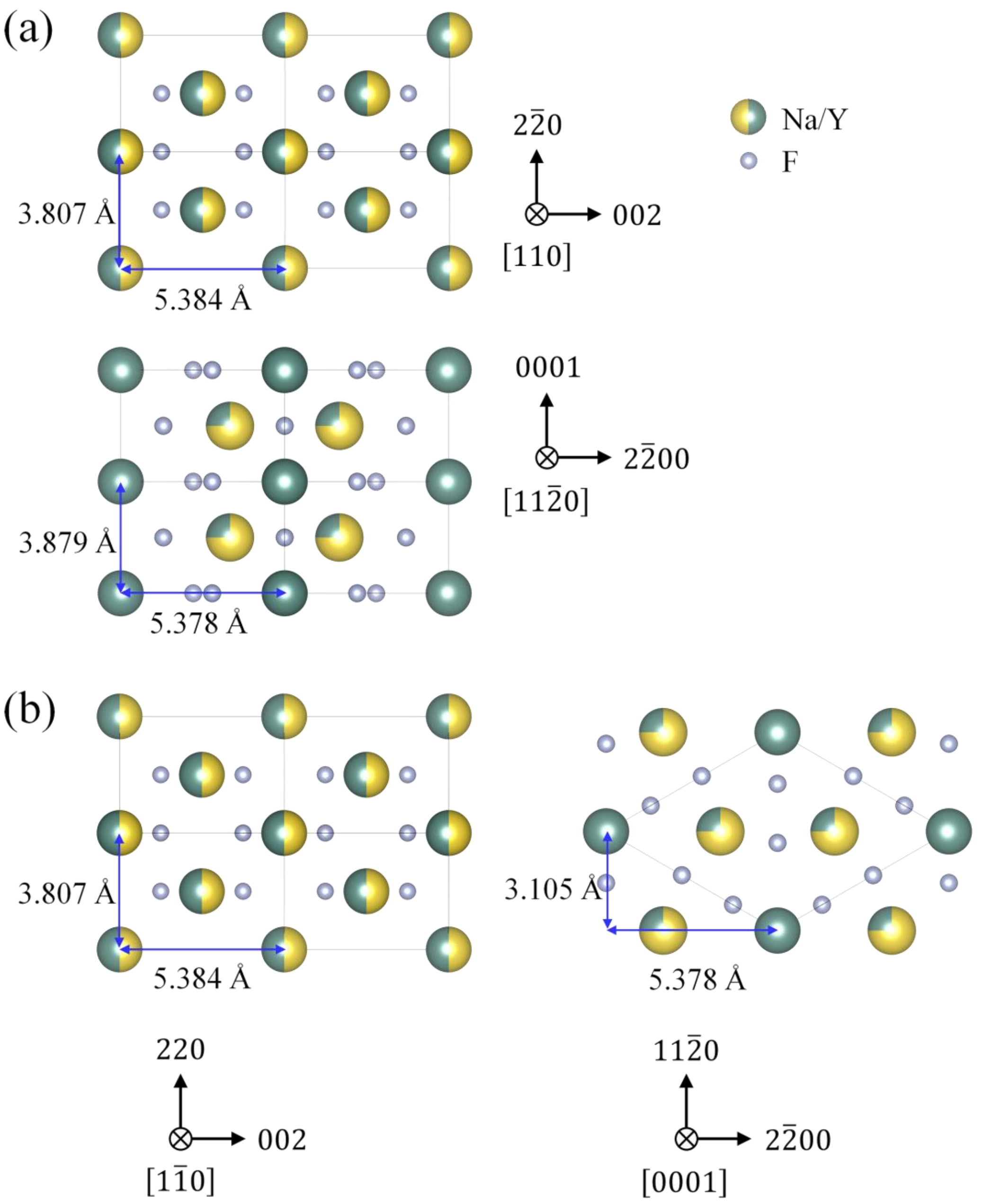

Figure S8. Schematic illustration of the atomic rearrangement of β- to α-phase transformation. (a) Atomic structures along the [110] and [11$\bar{2}$0] directions of the cubic and hexagonal structures, respectively. (b) Atomic structures along the [1$\bar{1}$0] and [0001] directions of the cubic and hexagonal structures, respectively.
